# The “play gap”: socioeconomic stratification in pediatric physical activity and its long-term health implications

**DOI:** 10.3389/fpubh.2026.1828942

**Published:** 2026-07-03

**Authors:** Shuangshuang Yan, Yanfang Liang

**Affiliations:** Qingdao Hengxing University of Science and Technology, Qingdao, China

**Keywords:** behavioral problem, children and adolescents, physical activity, public health, socioeconomic stratification, structural inequity

## Abstract

Physical inactivity in children and adolescents is not only a behavioral problem but also a manifestation of structural inequity. Across countries and within populations, opportunities for physical activity are unequally distributed by sex, socioeconomic position, disability status, neighborhood conditions, and access to supportive social and built environments. As a result, those with the greatest health needs often face the greatest barriers to participation. In this review, we synthesize recent evidence on the determinants of inequity in child and adolescent physical activity, assess the strengths and limitations of major policy levers, and examine emerging intervention strategies designed to improve reach, relevance, and sustainability. We argue that progress will depend on moving beyond universal but weakly implemented recommendations towards equity-oriented approaches that are context-sensitive, cross-sectoral, and accountable for implementation. Schools, financial support mechanisms, urban design, digital tools, and participatory models all hold promises. Still, their effects are constrained when policy ambition is not matched by delivery capacity or when interventions fail to address underlying social gradients. Future progress requires a shift from fragmented programmes to resilient systems that embed equity in design, prioritize disadvantaged groups, strengthen surveillance and evaluation, and position young people and communities as active partners in change.

## 1 Introduction

Globally, insufficient physical activity among children and adolescents has emerged as a pressing public health challenge. Substantial evidence indicates that regular physical activity yields multiple benefits for the physical, psychological, and social health of adolescents (1–3). However, the relevant recommendations set by the World Health Organization (4) are far from being universally adhered to among youth worldwide. Recent studies indicate that, despite national variations, global adolescent physical activity levels have generally plateaued at a concerningly low level over the past decade (5–7). Underlying this pervasive issue lies profound inequality. Participation in physical activity is not distributed equally; rather, it is strongly influenced by a complex array of social, economic, environmental, and policy factors, resulting in significant disparities in the opportunities to access and maintain an active lifestyle among adolescents of different genders, races, socioeconomic statuses, and ability levels (8, 9). The COVID-19 pandemic and its associated quarantine measures, acting as a natural experiment, further exposed and exacerbated these pre-existing inequalities, triggering a precipitous decline in the physical fitness of adolescents (10). The central public-health task has therefore shifted from simply “increasing activity” to the more demanding goal of “promoting equity”—ensuring that all young people, regardless of background, can access safe, accessible, and appealing opportunities to be active.

Throughout this mini-review, we distinguish equity from equality. Equality means providing the same resources or programmes to everyone; equity means allocating resources and tailoring opportunities according to need, so that the groups facing the greatest barriers receive proportionately greater support to reach comparable outcomes. The distinction is consequential: a single national guideline or an identical curriculum delivered to every school can leave existing gaps intact, or even widen them, because more advantaged groups are better placed to take up uniform offers. An equity-oriented approach instead prioritises disadvantaged groups and adapts the type, location, cost, and delivery of opportunities to context.

Against this backdrop, the mini-review integrates recent evidence to analyse the multidimensional determinants of inequity, appraise the effectiveness and limits of current strategies, and outline a more resilient, systemically equitable promotion framework. The key domains, representative evidence, and policy implications are summarised in Table 1.

**Table 1:** Key domains, representative evidence, and policy implications for reducing inequities in child and adolescent physical activity.

Domain	Representative evidence	Main finding	Equity relevance	Policy/practice implication
Global patterns and cross-national disparities	Bann et al. (8), Reilly et al. (7), van Sluijs et al. (3)	Across countries, child and adolescent physical activity remains persistently low, with marked variation by sex, socioeconomic position, and national policy context.	Average national estimates can obscure large within-country inequalities and unequal access to opportunities for activity.	Strengthen routine surveillance using disaggregated indicators and embed equity targets into national physical activity strategies.
Individual vulnerability and disability	Healy et al. (13), Brown et al. (17), Arnell et al. (18)	Adolescents with autism spectrum disorder show lower participation in physical activity and sport, alongside higher obesity risk; parental support is a major facilitator.	Universal programmes alone are insufficient for adolescents facing disability-related, sensory, or social participation barriers.	Adopt inclusive programme design, adapted delivery, and caregiver-supported participation pathways.
Family socioeconomic barriers	Mori et al. (9), Alliott et al. (14), Chzhen et al. (15)	Young people from less affluent households report fewer active days, fewer sports tried, and more barriers related to cost, transport, injury concerns, and feeling unwelcome.	Material disadvantage constrains not only participation frequency but also the diversity and continuity of activity opportunities.	Reduce user fees, subsidise participation, improve transport access, and design low-cost entry points for sustained engagement.
Peer, family, and school social environment	Lawler et al. (16), Foubister et al. (19), Carver et al. (45)	Peer support, family encouragement, and supportive school climates influence initiation, maintenance, and decline in physical activity.	Social support is unevenly distributed and can either buffer or amplify structural disadvantage.	Incorporate peer-led, family-engaged, and school-culture components rather than relying on curriculum alone.
Built environment, safety, and neighborhood conditions	Arroyo-Johnson et al. (20), Gu et al. (21), Addas et al. (22), Shams-White et al. (30)	Lower-resourced neighborhoods often have poorer playground safety, weaker maintenance, and lower perceived safety, all of which suppress physical activity.	Environmental deprivation compounds social disadvantage and disproportionately affects girls and minoritised groups in some settings.	Invest in safe, maintained, accessible recreational space, traffic calming, and neighborhood-level safety improvements.
COVID-19 as a stress test of inequality	Jurak et al. (10), Eyler et al. (23), Kovacevic et al. (42), Hasson et al. (41)	Pandemic restrictions triggered sharp declines in physical activity and exposed the fragility of existing support systems, especially for already vulnerable groups.	Crises magnify pre-existing inequities when activity opportunities depend on informal support, safe outdoor space, or school-based provision.	Build resilient promotion systems capable of rapid adaptation across school, home, and community settings.
School policy levers and the implementation gap	Thompson et al. (24), Sutherland et al. (25), Bengoechea et al. (26), Moore et al. (27), Belton et al. (40), Corder et al. (31)	Physical education and recess mandates are common, but accountability and dedicated funding are often absent; multicomponent whole-school models can help, although effects are not universal.	Implementation quality determines whether school policy narrows or reproduces inequality between advantaged and disadvantaged schools.	Pair legislation with funding, monitoring, implementation support, and whole-school system redesign.
Financial incentives and subsidy policies	Foley et al. (28)	The Active Kids voucher programme increased the number of days meeting activity guidelines across sociodemographic groups, demonstrating the promise of direct financial support.	Subsidies can reduce cost barriers, but unequal awareness, application capacity, or local programme availability may limit reach.	Use targeted vouchers or subsidies with uptake monitoring, outreach to underserved families, and alignment with community sport capacity.
Urban planning and place-based strategies	Pollack Porter et al. (29), Mindell et al. (32), Shams-White et al. (30)	Play Streets, active transport, and walkable environments can expand local opportunities for activity, particularly where permanent infrastructure change is difficult.	Spatial inequities in transport and public space access mean that design benefits are not evenly distributed.	Integrate public health, transport, and urban planning to prioritise under-resourced neighborhoods and safe active travel routes.
Technology-enabled and participatory approaches	Ng et al. (34), Ahn et al. (35); Wort et al. (36), Abraczinskas and Zarrett (37), Besenyi et al. (38)	Wearables, apps, and gamified tools can support motivation and monitoring, while participatory models such as YPAR empower youth to identify barriers and generate local solutions.	Technology can widen or narrow inequity depending on usability, access, school capacity, and whether youth voices shape intervention design.	Combine digital tools with co-design, teacher support, and youth participation rather than treating technology as a stand-alone solution.
**Mechanisms, future priorities, and economic rationale**	Beets et al. (33), Zhang et al. (39), Tamura et al. (43), Powell-Wiley et al. (44), Liu et al. (46)	Effective interventions often work by expanding, extending, or enhancing activity opportunities; social network mechanisms and intrinsic motivation matter, and reducing disparities yields substantial health and economic returns.	Equity-oriented action is justified not only ethically but also clinically and economically.	Future research should use objective surveillance, intersectional evaluation, implementation science, and mechanism-informed intervention design.

YPAR, Youth Participatory Action Research.

This table synthesises the main conceptual and empirical strands of the uploaded review and is designed as a narrative summary table rather than a full study-by-study extraction.

## 2 Review approach

As a mini-review, this is a narrative, theory-informed synthesis rather than a systematic review, and it does not claim to be exhaustive. To improve transparency, we searched PubMed, Web of Science, and Scopus for peer-reviewed, English-language articles published between January 2015 and February 2026, combining population, behavior, and equity search terms, and we hand-searched the reference lists of key articles. Priority was given to systematic reviews, large cross-national and longitudinal studies, natural experiments, and policy evaluations, with smaller studies retained when they usefully illustrated mechanisms or under-represented contexts. In synthesising this literature, we gave explicit weight to study design and to the consistency of findings across settings: large surveys, cohorts, natural experiments, and randomised controlled trials were treated as more robust than small, cross-sectional, or single-setting studies. Because most determinant studies are observational, we interpret their associations as correlational rather than causal unless a quasi-experimental or experimental design supports a stronger inference. Given marked heterogeneity, the evidence was synthesised narratively around the socio-ecological framework below rather than pooled, and no formal risk-of-bias assessment was undertaken.

## 3 Theoretical framework: a socio-ecological perspective

**Figure 1:**
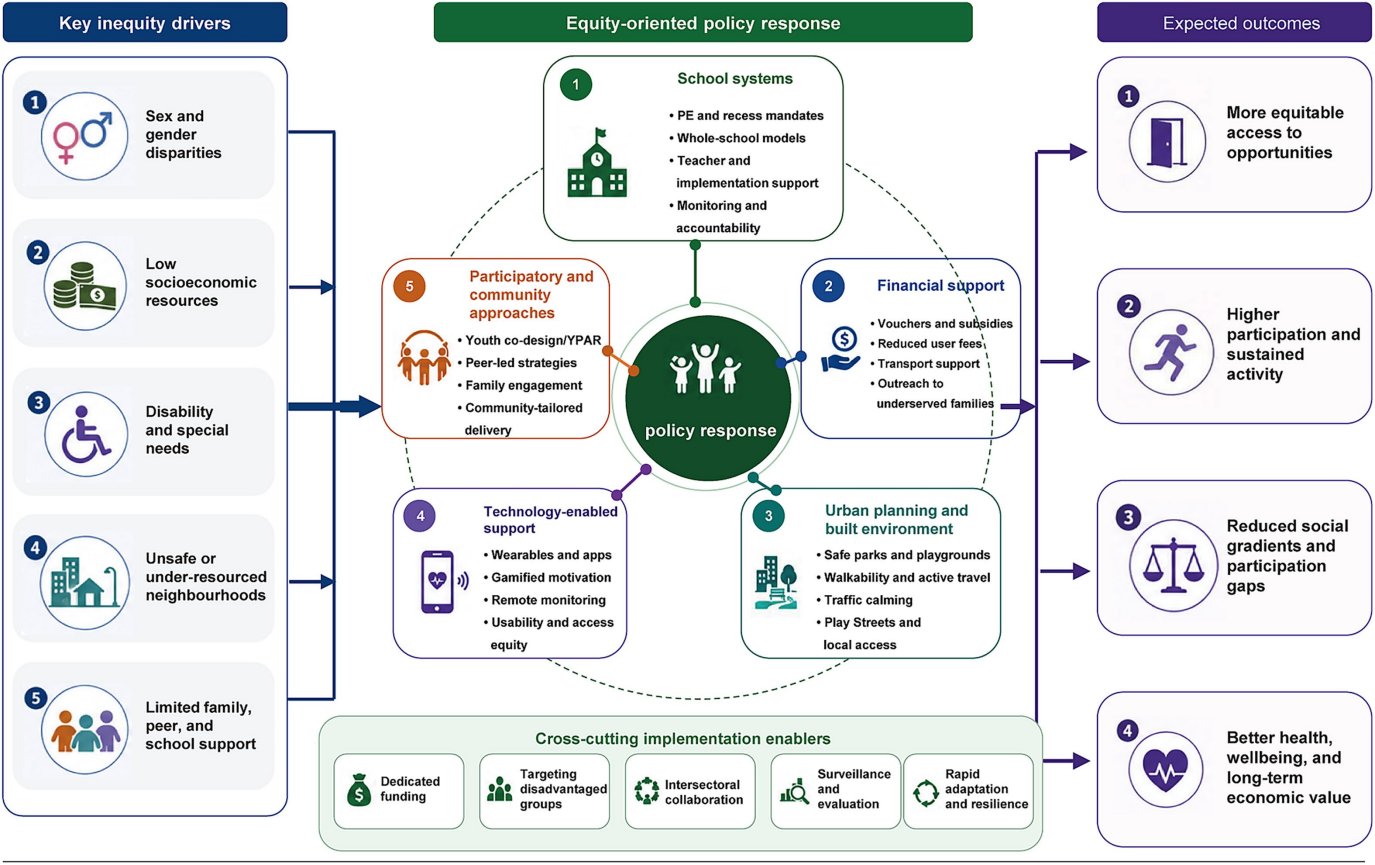
Conceptual framework for promoting equity in child and adolescent physical activity. Key inequity drivers operating across the levels of the socio-ecological model (left) are addressed through an integrated set of equity-oriented policy levers and intervention innovations (center; Sections 5, 6), underpinned by cross-cutting implementation enablers, in order to achieve more equitable participation and health outcomes (right).

To organise these heterogeneous determinants, the review is anchored in the socio-ecological model of health behavior, which builds on Bronfenbrenner’s ecological systems theory and its adaptation to health promotion (11, 12). Behavior is conceptualised as the product of reciprocal influences operating across nested levels: the individual (biological characteristics, disability, motivation, self-efficacy); the interpersonal or micro level (family, peers, and the immediate school setting); the community and physical-environment, or meso, level (neighborhood safety, the built environment, and access to facilities); and the macro level of policy, culture, and socioeconomic structure. These levels are interdependent, conditions at higher levels constrain or enable behavior at lower levels, and disadvantage tends to accumulate where deprivation coincides across several levels at once. A socio-ecological lens suits an equity analysis for two reasons. First, it makes explicit that unequal participation is rarely attributable to individual choice alone, but reflects the differential distribution of resources, safety, and opportunity across social groups. Second, it implies that durable change requires coordinated action at several levels simultaneously, an insight that underpins the calls for systems thinking and whole-school transformation discussed below. These nested, interdependent levels, and the accumulation of disadvantage across them, are represented on the left-hand side of Figure 1, which we use throughout as an organising device linking the drivers of inequity (Section 4) to the responses they require (Sections 5, 6).

## 4 Current status and multidimensional influencing factors of adolescent physical activity inequality from a global perspective

### 4.1 Macro level: cross-national variation and policy context

Inequality in adolescent physical activity is a multidimensional global phenomenon, with its manifestations and drivers varying significantly both within and between countries. At the macro level, a comparative study spanning 52 countries found widespread transnational variations in adolescent physical activity levels, which are closely associated with national physical education policies, as well as gender and socioeconomic disparities (8). At the meso and micro levels, these inequalities interact and become entrenched through various tiers of the socio-ecological model.

### 4.2 Individual level: health status and disability

Individual characteristics and health status constitute the foundational level. For instance, compared to their neurotypical peers, adolescents with autism spectrum disorder exhibit significantly lower participation rates in moderate-to-vigorous physical activity, light physical activity, and sports, alongside a higher prevalence of overweight or obesity (13). This suggests that certain groups face higher barriers to participation due to their intrinsic conditions.

### 4.3 Family socioeconomic position

Family socioeconomic status is another core determinant. Research consistently demonstrates that adolescents from less affluent families report fewer active days, fewer types of sports tried, and lower rates of sports participation (9, 14). They more frequently report barriers such as prohibitive costs, lack of transportation, fear of injury, or feeling unwelcome on teams (9). This socioeconomic gradient has been validated across multiple countries, with adverse health behaviors often clustering in less affluent households (15).

### 4.4 Interpersonal level: peer, family, and school social environments

The social environment, particularly peer and family support, plays a pivotal role. Peer support and role modeling are crucial predictors for the maintenance, initiation, or cessation of physical activity among adolescents (16). Parental supportive behaviors, including encouragement and co-participation, are positively correlated with the physical activity levels of adolescents with autism spectrum disorder (17). However, parents may also require external support to facilitate their adolescents’ activity participation due to the perceived daunting nature of the task (18). As a vital social setting, the school’s social environment is associated with changes in adolescents’ physical activity (19).

### 4.5 Physical environment: built environment and neighborhood safety

The built environment and neighborhood safety constitute the physical context influencing physical activity. However, the distribution of these resources is highly unequal. A study on public playgrounds in St. Louis, USA, found that neighborhoods with higher proportions of adolescents and African American residents scored significantly lower in overall playground safety and maintenance (20). This disparity in perceived safety directly impacts behavior. During the COVID-19 pandemic, perceived neighborhood safety influenced changes in physical activity either directly or indirectly by affecting adolescents’ ability to cope with restrictions (21). Low-socioeconomic status and Hispanic adolescents reported significantly lower perceived safety for walking or playing in their neighborhoods compared to their mid-to-high-socioeconomic status non-Hispanic peers, and they experienced a steeper decline in moderate-to-vigorous physical activity (21). Research in Saudi Arabia also highlighted the critical impact of neighborhood safety, particularly traffic management and accessibility, on the physical activity behaviors of adolescents, especially females (22).

### 4.6 COVID-19 as a stress test of inequity

The COVID-19 pandemic, as a major shock, underscored the resilience of these unequal structures. Quarantine measures led to an overall decline in physical activity levels among children and adolescents, with social barriers (e.g., lack of playmates, absence of adult supervision) explaining the reduction in activity more significantly than environmental barriers (e.g., insufficient space) (23). Long-term health gains can be eroded in the short term; data from Slovenia revealed that merely 2 months of self-isolation offset the achievements the country had strived for over a decade through national public health policies and interventions (10).

In summary, the inequality in adolescent physical activity is the result of the intertwined effects of multiple factors, including individual health status, family socioeconomic status, social support networks, and unequal built environments. These factors mutually reinforce one another, rendering vulnerable groups even more susceptible during crises like the pandemic. Understanding this complex web is a prerequisite for designing effective and equitable interventions and policies.

## 5 Key policy levers and implementation challenges in promoting child and adolescent physical activity

Figure 1 provides an integrated overview of how the key drivers of physical-activity inequity (Section 4) can be addressed through a coordinated set of equity-oriented policy and intervention responses (Sections 5, 6), supported by cross-cutting implementation enablers, to achieve more equitable participation and health outcomes. Confronted with pervasive inequality, policy interventions are viewed as crucial levers to reverse the situation and foster supportive environments. Effective policies can systemically expand opportunities, lower barriers to entry, and provide a framework for sustainable change. Currently, school policies, financial incentives, and urban planning are three policy domains garnering significant attention and accumulating substantial evidence, yet each faces unique implementation challenges (3, 6).

Schools, where adolescents spend the majority of their time, are natural focal points for policy intervention. Many countries have enacted legislation mandating physical education classes and recess to ensure baseline activity opportunities for youth. However, a vast chasm exists between policy enactment and effective enforcement. A recent review of state-level laws in the United States found that while the vast majority of states have physical education laws and nearly half have recess laws, these mandates rarely include accountability provisions and almost entirely lack dedicated funding (24). The absence of accountability and funding is widely recognized as the primary obstacle to effective enforcement. Successful school interventions require moving beyond a single curriculum to adopt a whole-school approach. For example, the “Physical Activity for Everyone” intervention effectively mitigated the decline in physical activity among adolescents from disadvantaged schools through multicomponent strategies (25). Nevertheless, achieving whole-school transformation requires systems thinking. Researchers suggest that future school-based physical activity promotion should employ a systems approach, maximizing partnership actions and leveraging policies, while acknowledging the comprehensive cognitive, emotional, and social benefits of physical activity from a strengths-based perspective (26). Implementing comprehensive whole-school physical activity programs is inherently complex, necessitating the application of implementation science frameworks to guide the “how-to,” involving organizational characteristics, implementation processes, and provider attributes (27).

Financial incentive policies aim to directly reduce the economic burden of sports participation for families. The “Active Kids” voucher program implemented in New South Wales, Australia, serves as a large-scale natural experiment. The program provided vouchers for school-aged children registered in structured sporting activities. Evaluations indicated that with the use of vouchers, the number of days children met physical activity guidelines increased from 4.0 to 4.9 days per week, with activity increases observed across all socio-demographic groups (28). This program demonstrated the potential of financial subsidies to increase child and adolescent sports participation at the population level. However, issues regarding its long-term cost-effectiveness, equitable accessibility across different socioeconomic groups (e.g., whether all families are aware of and can easily apply for it), and alignment with community sports provision capacity require ongoing attention.

Urban planning and public space policies can fundamentally shape the physical activity environments of adolescents. Integrating health equity into urban design means ensuring all neighborhoods, especially under-resourced ones, possess safe, convenient, and attractive recreational spaces. The “Play Streets” initiative in Chicago is an innovative place-based intervention that temporarily closes streets to create safe, free spaces for youth to be active and access community resources (29). Such temporary, low-cost, and highly flexible interventions offer a viable pathway to promoting health equity in cities where modifying the built environment is difficult. Another strategy involves enhancing neighborhood walkability and safety; research shows that adolescents living in neighborhoods with higher population density and older housing (typically associated with walkability) exhibit higher levels of moderate-to-vigorous physical activity, though this association is inconsistent across neighborhoods of varying socioeconomic statuses (30). This suggests that adolescents in low-socioeconomic status neighborhoods may require additional environmental supports and intervention opportunities beyond mere neighborhood walkability.

Despite these potent policy levers, their successful implementation encounters universal challenges. First, aligning policy design with local contexts is paramount. An intervention effective in one country or cultural setting may not be directly transferable to another (31). Second, cross-sectoral collaboration is both a bottleneck and a key to success. Promoting adolescent physical activity involves multiple sectors, including education, health, sports, urban planning, and transportation; breaking down silos and forging policy synergy is crucial (32). Third, the sustainability and scalability of interventions pose a major challenge. Many research-based projects demonstrate significant effects on a small scale, but their efficacy often diminishes when scaled up or implemented in real-world conditions. Finally, and most centrally, is the challenge of ensuring that the policy itself and its implementation do not exacerbate, but rather mitigate, existing inequalities. This demands that policymakers adopt an acute equity lens, proactively identifying and responding to the differentiated needs of various groups throughout the policy design, implementation, and evaluation processes.

## 6 Equity-targeted intervention strategy innovations and practical evidence

Within the policy framework, intervention strategies tailored to different contexts and specific populations are continuously innovating and evolving. The core objective of these strategies is to transition from a “one-size-fits-all” model to more targeted, empowering, and inclusive approaches to effectively narrow disparities in physical activity participation. Recent research highlights several distinct trends in strategy innovation and evidence accumulation (33).

Strategy innovation is primarily reflected in leveraging technology for empowerment and enhancing participatory engagement. Wearable devices and mobile applications offer new tools for monitoring, feedback, and motivation. Studies indicate that adolescents using activity tracker applications show a positive correlation between physical activity behaviors and the use of these tools (34). Virtual pets developed based on the Youth Physical Activity Promotion Model framework can significantly increase children’s daily physical activity time through goal-setting and reward mechanisms, functioning by enhancing self-efficacy and beliefs regarding physical activity (35). However, the successful application of technology relies on optimal user experience and system support. Introducing wearable technology in school settings requires considering teacher burden, data interpretation, and how to seamlessly integrate the technology into existing pedagogical practices (36). A more transformative innovation is Youth Participatory Action Research (YPAR). This method engages adolescents, particularly marginalized youth, as co-researchers of their own community activity environments, utilizing methods like Photovoice to identify barriers and propose solutions. Practice has proven that this approach not only enhances adolescents’ personal sense of empowerment but also facilitates tangible changes at the community level, such as increasing sports opportunities for girls, often yielding unique, youth-generated solutions overlooked by adult-led interventions (37). The development and application of electronic community park audit tools also demonstrate how mobile technology can assist youth in participating in community environment assessments and advocacy, driving policy, systems, and environmental changes conducive to health (38).

Secondly, intervention design places greater emphasis on theory-driven approaches and mechanism exploration. The “Theory of Expanded, Extended, and Enhanced Opportunities” proposed by Beets et al. (33) provides a concise and powerful framework for understanding how interventions work. This theory posits that many effective interventions essentially operate by creating new activity opportunities, extending the duration of existing ones, or enhancing their quality. This prompts researchers to focus more on these fundamental mechanisms of change rather than merely on complex individual behavioral constructs. Social network analysis provides a refined tool for understanding the critical mechanism of peer influence. By analyzing the structure of adolescents’ social networks, researchers can more accurately identify the processes of peer influence and peer selection, thereby designing more effective peer-based intervention strategies (39).

Regarding practical evidence, research is dedicated to answering the core question: “What interventions work for whom, and in what contexts?” For adolescents with autism spectrum disorder, family-level interventions show promise; parental behavioral regulation strategies (e.g., goal setting, planning) and supportive behaviors are key pathways to promoting their children’s physical activity (17, 18). For adolescents of lower socioeconomic status, interventions need to transcend neighborhood walkability to provide more diversified activity opportunities and environmental support (30). Schools remain the primary arena for implementing interventions, but successful school-based interventions must be comprehensive and systemically supported. For instance, the Y-PATH whole-school intervention targeting Irish adolescents demonstrated positive effects in mitigating the decline of physical activity among girls during a 24-month follow-up (40). However, not all well-designed interventions achieve their anticipated outcomes. The GoActive cluster randomized controlled trial in the UK found that although the peer-led school intervention was well-received by students and teachers, it failed to effectively prevent the age-related decline in adolescent physical activity levels after 10 months (31). This outcome highlights the challenges of implementing complex interventions in real-world educational settings and underscores the necessity for continuous evaluation and adaptation.

Furthermore, the transferability and adaptability of interventions were tested during crises like the COVID-19 pandemic. Research indicates that the rapid adaptation of school-based physical activity interventions to home environments was crucial for safeguarding activity opportunities for youth, especially high-risk groups, during lockdowns (41). This capacity for rapid adaptation is itself an essential component of the resilience of future intervention systems. Concurrently, shifts in physical activity cognitions during the pandemic offered new insights: despite an overall decline in behavior, adolescents’ application of behavioral regulation skills increased, and pre-pandemic activity habits emerged as the strongest cognitive predictor of activity levels during the pandemic (42). This emphasizes the long-term value of cultivating robust physical activity habits during normal times.

## 7 Conclusion and future prospects: building an equitable, resilient, and systemic future for physical activity promotion

The field of child and adolescent physical activity promotion is at a critical juncture of transformation. Substantial evidence demonstrates that physical inactivity and its accompanying inequalities constitute a complex systemic puzzle, deeply rooted in broad social, economic, environmental, and policy structures. Consequently, fragmented interventions focusing solely on individual behavior change yield limited and unsustainable results. Looking to the future, building an equitable, resilient, and systemic physical activity promotion ecosystem requires a comprehensive overhaul from conceptualization to action.

First, an action agenda centered on equity must be established. Research has clearly delineated patterns of inequality based on gender, socioeconomic status, race, ethnicity, ability status, and residential location (21, 43). Future policy and intervention designs must explicitly target the elimination of these disparities. This requires adopting an intersectional perspective to understand how compounding marginalized identities exacerbate barriers to activity. Economic modeling studies provide robust support for this, showing that eliminating socioeconomic and gender disparities in US adolescent physical activity would not only yield immense health benefits but also generate significant economic returns; the averted healthcare costs and productivity losses far exceed the potential costs of program investment (43, 44). This furnishes policymakers with a compelling economic rationale for investing in equity promotion.

Second, embrace systems thinking and cross-sectoral synergy. Adolescent physical activity behaviors are mutually influenced by multilevel factors across family, school, community, and policy domains. Efforts from any single sector are insufficient to resolve this systemic issue. Within the school environment, its role must be reimagined, adopting a whole-school, systemic approach to deeply embed physical activity into the school’s culture, policies, and daily practices, rather than treating it as an add-on task. Beyond the school, effective collaboration mechanisms must be established among sectors such as education, public health, urban planning, transportation, sports, and community organizations. For instance, through policy coordination, public transit design can be integrated with school commuting needs to provide youth with safe, affordable active transport options (32). Urban planning should prioritize the construction and maintenance of safe, accessible recreational spaces in under-resourced communities (20, 29).

Third, invest in innovative research, surveillance, and adaptive learning. Future research needs to fill critical gaps. In terms of measurement, there should be greater use of objective devices combined with systemic social network analysis to more accurately delineate activity patterns, environmental exposures, and social influence mechanisms (45). Regarding interventions, research on implementation processes, contextual factors, and mechanisms of action should be strengthened to understand “why it works” and “under what conditions it works” (33). Particular attention must be directed toward conducting research in low- and middle-income countries and rapidly urbanizing regions, where evidence is relatively scarce yet the need is urgent (46). Continuous national surveillance systems, such as SLOfit in Slovenia, possess irreplaceable value for tracking trends, evaluating policy impacts, and providing timely early warnings during crises (10).

Finally, empower youth and communities to build resilience. Transforming adolescents from passive recipients of interventions into active agents of change is critical for sustainable promotion. Utilizing participatory approaches to engage youth in identifying barriers and assets within their communities, and involving them in advocacy and solution design, not only generates interventions better tailored to their needs but also cultivates their health advocacy skills and civic consciousness (37). Meanwhile, constructing a resilient promotion framework means it can withstand external shocks like the pandemic. This demands that intervention designs possess flexibility and adaptability, function effectively across different settings, and assist adolescents in establishing solid physical activity habits and intrinsic motivation, enabling them to maintain the core elements of an active lifestyle even during times of upheaval (46).

### 7.1 Strengths and limitations

Several limitations should be acknowledged. As a mini-review, this synthesis is narrative rather than systematic; despite a structured search, it cannot be considered exhaustive, no formal quality appraisal or risk-of-bias assessment was undertaken, and the restriction to English-language publications may have introduced selection bias. More importantly, the studies synthesised here were conducted in diverse countries and sociocultural contexts, ranging from high-income settings such as the United States, Australia, Ireland, and Slovenia to rapidly urbanising and middle-income regions such as Saudi Arabia and China, in which the meaning, measurement, social patterning, and determinants of physical activity differ substantially. Findings obtained in one setting, therefore, cannot be assumed to transfer directly to another, and the inequities described here should be read as context-dependent patterns rather than universal effects. Evidence remains particularly scarce from low- and middle-income countries, which further constrains the generalisability of current conclusions and the strength of any cross-national inference. We have tried to mitigate these constraints by organising the evidence within an explicit socio-ecological framework, by signaling where findings are setting-specific, and by drawing comparative conclusions cautiously. Nonetheless, these limitations reinforce our central argument: equity-oriented action must be locally grounded, and future work should prioritise context-sensitive evaluation, harmonised and objective measurement, and broader geographical coverage, especially in under-represented regions—before firm generalisations are drawn.

In conclusion, the future path for promoting child and adolescent physical activity must inherently be one committed to health equity, reliant on systemic transformation, empowering to community youth, and continuously learning based on robust evidence. Only through resolute, synergistic, and sustained efforts can we ensure that all adolescents possess an equal opportunity to enjoy an active life and realize their health potential.
